# Rhinovirus-induced basic fibroblast growth factor release mediates airway remodeling features

**DOI:** 10.1186/2045-7022-2-14

**Published:** 2012-08-21

**Authors:** Chrysanthi L Skevaki, Stelios Psarras, Eleni Volonaki, Harris Pratsinis, Irini S Spyridaki, Mina Gaga, Vassiliki Georgiou, Stylianos Vittorakis, Aurica G Telcian, Paraskevi Maggina, Dimitris Kletsas, Dimitrios Gourgiotis, Sebastian L Johnston, Nikolaos G Papadopoulos

**Affiliations:** 1 UPC Research Laboratories, Allergy Department, 2nd Pediatric Clinic, University of Athens, 41 Fidipidou str, Athens, 115 27, Greece; 2 Biomedical Research Foundation, Academy of Athens, Athens, Greece; 3 Institute of Biology, NCSR “Demokritos” Ag. Paraskevi, Athens, Greece; 4 Sotiria” Athens Chest Hospital, Athens, Greece; 5 Imperial College London, London, UK

**Keywords:** Airway remodeling, Asthma, BFGF, Bronchial epithelium, Rhinovirus

## Abstract

**Background:**

Human rhinoviruses, major precipitants of asthma exacerbations, induce lower airway inflammation and mediate angiogenesis. The purpose of this study was to assess the possibility that rhinoviruses may also contribute to the fibrotic component of airway remodeling.

**Methods:**

Levels of basic fibroblast growth factor (bFGF) mRNA and protein were measured following rhinovirus infection of bronchial epithelial cells. The profibrotic effect of epithelial products was assessed by DNA synthesis and matrix metalloproteinase activity assays. Moreover, epithelial cells were exposed to supernatants from cultured peripheral blood mononuclear cells, obtained from healthy donors or atopic asthmatic subjects and subsequently infected by rhinovirus and bFGF release was estimated. bFGF was also measured in respiratory secretions from atopic asthmatic patients before and during rhinovirus-induced asthma exacerbations.

**Results:**

Rhinovirus epithelial infection stimulated mRNA expression and release of bFGF, the latter being positively correlated with cell death under conditions promoting rhinovirus-induced cytotoxicity. Supernatants from infected cultures induced lung fibroblast proliferation, which was inhibited by anti-bFGF antibody, and demonstrated increased matrix metalloproteinase activity. Rhinovirus-mediated bFGF release was significantly higher in an *in vitro* simulation of atopic asthmatic environment and, importantly, during rhinovirus-associated asthma exacerbations.

**Conclusions:**

Rhinovirus infection induces bFGF release by airway epithelium, and stimulates stroma cell proliferation contributing to airway remodeling in asthma. Repeated rhinovirus infections may promote asthma persistence, particularly in the context of atopy; prevention of such infections may influence the natural history of asthma.

## Background

Structural changes in the asthmatic lung, collectively known as airway remodeling, are now widely recognized and potentially associated with bronchial hyperresponsiveness, the incomplete therapeutic effect of corticosteroids, as well as the progressive decline of pulmonary function in asthmatic patients with more severe and chronic disease. Increased myofibroblast proliferation and subepithelial collagen deposition, wall thickening and angiogenesis are important components of airway remodeling [1]. Numerous studies pinpointed the central role of the bronchial epithelium in these processes [2], exemplified by its ability to release potent modulators of collagen turnover and fibroblast proliferation, including cytokines, growth factors, as well as matrix metalloproteases (MMP) [3-5].

The multifunctional basic fibroblast growth factor (bFGF or FGF-2) is a potential mediator of airway remodeling due to its ability to regulate migration and/or proliferation of vascular endothelial cells, fibroblasts, airway smooth muscle cells and myofibroblasts [6]. Indeed, bFGF is increased in the bronchoalveolar lavage (BAL) fluid of patients with asthma and further up-regulated upon allergen challenge [7]. Furthermore, the increased vasculature in asthmatic bronchial mucosa correlates with the number of cells expressing bFGF [8]. The asthmatic epithelium is a major source of bFGF [9]. Chemical or mechanical insult to the epithelium may lead to proliferation of myofibroblasts and increased collagen production due to the action of growth factors including bFGF [10]. Viral respiratory tract infections trigger acute asthma exacerbations, human rhinoviruses (RV) being the most frequent agent [11]. RVs infect and replicate in the lower airway epithelium [12], leading to local and systemic immune responses and production of inflammatory mediators [13], that have the potential of inducing and/or regulating airway remodeling [14]. We have recently demonstrated that RV infection may promote airway remodeling by inducing angiogenesis through vascular endothelial growth factor (VEGF) production by the bronchial epithelium [15].

Based on the above, we hypothesized that RV infection may also contribute to the fibrotic component of airway remodeling through bFGF, which may induce fibroblast proliferation and regulate collagen turnover. We assessed the ability of RV to stimulate bFGF release by human bronchial epithelium *in vitro*, evaluated the impact of the released bFGF on pulmonary fibroblast proliferation and measured the activity of MMP in supernatants of RV-infected bronchial epithelial cells. Moreover, we estimated the influence of the atopic asthmatic environment on bFGF release, as well as the abundance of bFGF *in vivo* during RV-associated asthma exacerbations.

## Methods

### Cell cultures

Human bronchial epithelial cells (BEAS-2B) (ECACC, Salisbury, UK) were grown as described [12,13]. Normal human bronchial epithelial (NHBE) cells were obtained from Clonetics, Wokingham, UK and derived from normal non-smoking adult donors. Primary human bronchial epithelial (PHBE) cells were derived from an adult volunteer without asthma after informed written consent and approval by the ‘Sotiria’ Hospital Review Board for Human Studies. PHBE and NHBE cells were grown in bronchial epithelial basal medium (BEBM), which was supplemented with growth supplements as recommended by the manufacturer, and they were used at passages 2–3.

Primary cultures of normal human lung fibroblasts were developed using the explant method [16], from apparently normal areas of the lungs of consenting volunteers undergoing surgery [17]. The human lung fibroblast strain CCD19Lu was purchased from ECACC. All fibroblasts were routinely cultured in Minimal Essential Medium (MEM) supplemented with 10% Fetal Bovine Serum (FBS). Primary cultures were used between passages 3 and 6. Harvesting by trypsinization and cell counting were performed as previously described [16]. All cells were tested periodically and were found to be mycoplasma-free.

### Virus cultures and titration

Major and minor rhinoviruses (RV16 and RV1b, respectively) were propagated in Ohio-HeLa cells (ECACC) at 33°C in a humidified 5% CO2 incubator, as previously described [12]. Briefly, upon development of full cytopathic effect (CPE), cells and supernatants were harvested, frozen and thawed, clarified by centrifugation, aliquoted and stored at −70°C. Lysates of parallel Ohio-HeLa cell cultures, not infected with virus, were used as controls in subsequent experiments. In order to determine RV titres, Ohio-HeLa cells were seeded in 96-well plates until 60-70% confluence at the time of infection. Logarithmic dilutions of RVs were made in multiple wells and the plates were fixed and stained after five days with 5% formaldehyde, 5% ethanol and 0.1% crystal violet in PBS. The end-point titer was defined as the highest dilution at which a CPE was detected in at least half of the wells and expressed as the inverse logarithm of this dilution.

### Epithelial cell infection and collection of conditioned media (CM)

Low passage (10–19) BEAS-2B cells were grown and infected by RV1b as described [12,13], at multiplicity of infection (MOI) of 1, unless otherwise specified. For the fibroblast proliferation assay, BEAS-2B cells were infected with RV1b under serum-free conditions, in order to eliminate any direct effect of the serum contained in supernatants on the proliferation of the stroma cells. For the experiments involving exposure of BEAS-2B cells to an atopic environment, we used pooled supernatant from peripheral blood mononuclear cells (PBMC), which were obtained by healthy donors and atopic asthmatic subjects and infected by RV1b in vitro in the course of a recently published study, as described [18]. Briefly, 0.6 mL of PBMC supernatant was added per well of epithelial cells and left for 24 h at 37°C, at which time it was removed and BEAS-2B were infected with RV1b at MOI 1.

Supernatants (conditioned media, CM) were collected 48 h after infection (unless otherwise specified), clarified by centrifugation (10 min/3000 g/4°C), and stored at −70°C until used in immunoassays. Control CM were collected from parallel cultures exposed to heat-inactivated RV1b (1 h at 58°C), ultraviolet radiation (UV)-inactivated RV1b (4 cm from a 100 W UV light source for 8 min) or HeLa cell lysates. Heat and UV inactivation of RV1b was effective as assessed by titration assays. Moreover, in some experiments, an anti-human VEGF antibody (R&D Systems, Europe) was added to BEAS-2B after RV1b infection at final concentrations of 5 and 0.1 μg/ml. For the DNA synthesis assay, CM were UV irradiated in order to inactivate possible residual RV1b, and thus eliminate any direct RV1b effect on fibroblast proliferation. UV irradiation did not result in significant alterations on bFGF levels as assessed by ELISA performed before and after UV irradiation of CM.

### Cytotoxicity assay

RV-induced cytotoxicity on BEAS-2B cells was measured using a colorimetric assay, as previously described [19]. Epithelial cells were plated in 48-well plates in serial dilutions and allowed to grow for 48 h, reaching confluence of 100%, 50%, 25% and 12,5%. 48 h after incubation with RV1b or control medium, cells were washed twice in PBS and a volume of crystal violet staining buffer equal to the 1/5th of the original culture medium was added to the wells as indicator of cell viability [20,21].

Cells were incubated for 30 min at room temperature followed by extensive washing with distilled water. After air drying, 0.2 ml of a destain buffer (16.6% v/v glacial acetic acid, 50% v/v methanol in ultra pure water) was added to the wells for 5 min. Cells were fully destained and the produced color was transferred to a clear 96 well ELISA plate and optical density was measured with a photometer at 595 nm (Ceres 90°C, Bio-Tec Instruments, Inc, Winooski VT, USA) [12]. Cytotoxicity was estimated as % of the negative control (1- O.D RV infected/ O.D HeLa * 100).

### DNA synthesis assay

The effect of epithelial cell CM on human lung fibroblast proliferation was estimated using tritiated thymidine incorporation into newly synthesized DNA, essentially as described [22]. Medium supplemented with 2 ng/ml human recombinant bFGF (R&D Systems Europe) served as a positive control. In order to neutralize bFGF-activity, media conditioned by epithelial cells or media supplemented with recombinant bFGF were preincubated with a goat anti-bFGF neutralizing antibody (R&D Systems Europe) at a final concentration of 10 μg/ml for 60 min at 37°C. Control experiments with total goat IgG indicated no interference with this DNA synthesis assay (data not shown).

### MMP activity assay

Conditioned media derived from RV1b-infected or control epithelial cells were mixed at a 1:1 ratio with MMP-incubation buffer 50 mM Tris (pH: 7.6), 150 mM NaCl, 10 mM CaCl_2_, 1 μM ZnCl_2_ containing the fluorogenic substrate Dabcyl-Gaba-Pro-Gln-Gly-Leu-Glu-(EDANS)-AIa-Lys-NH_2_ (TNO211, Calbiochem) at a final concentration 7 μM [23]. This peptide contains the MMP-cleavable bond Gly-Leu, as well as the fluorophore EDANS, whose fluorescence is quenched by the close proximity of Dabcyl. After enzymatic cleavage the quenching is eliminated and fluorescence is detectable. The catalytic efficiency of TNO211 is very high for MMP-9 (kcat/Km = 619,000 M-1 s-1), MMP-2 (kcat/Km = 206,000 M-1 s-1), MMP-3 (kcat/Km = 40,000 M-1 s-1), and MMP-1 (kcat/Km = 21,000 M-1 s-1) [23]. In the experiments presented herein, after incubation of the TNO211-supplemented conditioned media at 37°C in the dark for 3 h, fluorescence emission was counted at 480 nm after excitation at 340 nm, using a Fluostar Galaxy microplate reader (BMG Labtechnologies, Offenburg, Germany)

### Nasopharyngeal wash samples

Samples from 10 atopic children (6 boys, mean age 10.7 years, range 8–16) with intermittent or mild persistent asthma according to the GINA guidelines and positive skin prick tests (SPT) to at least 1 of a panel of locally relevant allergens, obtained in the course of a prospective clinical study conducted at the “P&A Kyriakou” Children’s Hospital and approved by the hospital’s Review Board, had been collected after informed written consent of surrogates at a symptom-free period and subsequently during an RV-associated asthma exacerbation. The presence of RV (and its absence at baseline), as well as the absence of respiratory syncytial virus (RSV), corona, adeno-, influenza A and B and parainfluenza viruses in the nasopharyngeal washes during the exacerbations was assessed by RT-PCR [24].

### ELISA assays

Levels of bFGF were measured in cell supernatants using a commercially available sandwich ELISA assays (R&D Systems, Europe) according to the manufacturer’s instructions. The lower detectable level was 3 pg/ml. Nasopharyngeal wash samples were treated with heparin in order to increase release from heparin sulphate binding sites as described in Shute et al. [9]. Briefly, heparin (Sigma Chemical Co) was added to the samples at a final concentration of 100 μg/ml and samples were incubated for one hour at 37°C. Following incubation, bFGF that had been released and captured by the primary antibody was quantified by completing the subsequent ELISA steps according to the manufacturer’s instructions.

### RT-PCR analysis

cDNA was prepared from RV1b-infected and HeLa lysate-exposed BEAS-2B cells, which were infected under conditions not allowing virus mediated cytotoxicity, ie high cell culture density. Subsequently, cDNA was subjected to semiquantitative RT-PCR against b-actin as applied in our previous work focusing on RV-mediated VEGF production [15]. bFGF-specific PCR primers were used (5^′^-GCC-TTC-CCG-CCC-GGC-CACTTC-AAG-G-3 and 5^′^-GCA-CAC-ACT-CCT-TTG-ATAGAC-ACA-A-3^′^) and the predicted amplicon length was 180 bp.

### Data and Statistical analysis

Distribution normality was assessed by the Kolmogorov-Smirnov test. Data are expressed as means ± SEMs. Comparisons between two groups were performed with independent sample t-test. In the case of more groups, one-way ANOVA with Bonferroni’s correction for subsequent between-group effect evaluation was used. A Wilcoxon signed-rank test was performed for comparing bFGF levels in nasopharyngeal secretions of children with atopic asthma at baseline to the respective levels observed during an RV-associated asthma exacerbation. Statistical analysis was conducted with Graph Pad Prism 3.0 software.

## Results

### RV infection stimulates bFGF release by human bronchial epithelial cells

**Figure 1:**
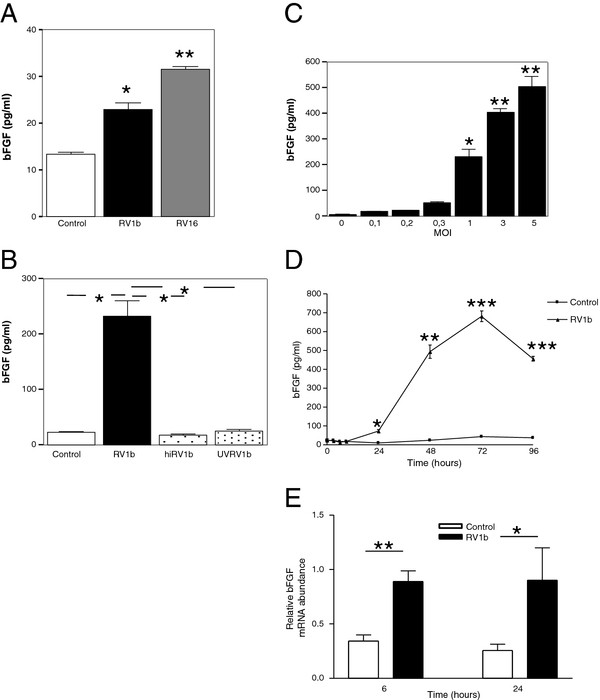
**Rhinovirus (RV) infection induces bFGF release and mRNA expression in human bronchial epithelial cells. A**, bFGF release by normal human bronchial epithelial (NHBE) cells exposed to RV1b or HeLa lysate (control); *p < .01, **p < .0001. **B**, bFGF release by BEAS-2B exposed to RV1b, heat-inactivated RV1b (hiRV), UV-inactivated RV1b (UVRV1b) or HeLa lysate (control); *p < .001. **C**, bFGF release by BEAS-2B infected by RV1b at different MOIs; * p < .01, ** p < .001. **D**, Time course of bFGF release on infection by RV1b. * p < .01, ** p < .001, *** p < .0001. For **A**, **C** and **D**, comparisons were made with cultures exposed to control medium. **E,** Relative abundance of bFGF mRNA in comparison to β-actin after RV1b infection of BEAS-2B; *p < .05; **p < .01. n ≥ 3 independent experiments.

Infection of NHBE cells with RV1b or RV16 at MOI 1 resulted in a significant induction of bFGF levels compared to control (Figure 1A; p < .01 for RV1b and p < .0001 for RV16). Similar results were obtained with PHBE cells (data not shown).

We next used RV1b to infect BEAS-2B cells, a bronchial epithelial cell line extensively served as a well-characterized in-vitro lower respiratory epithelium model of RV infection [13,15,19]. Infection of BEAS-2B cells resulted in a considerable (~10-fold) up-regulation of bFGF release (233.3 ± 27.4 versus 23.1 ± 1.4 pg/ml for control cultures; p < .001) by 48 h (Figure 1B). Exposure of BEAS-2B cells to heat- or UV-inactivated RV1b did not result in bFGF induction, suggesting that the effect was virus-specific (Figure 1B; p < .001). Furthermore, RV1b was shown to up-regulate bFGF release in a dose-dependent manner (Figure 1C). Time-course experiments showed that bFGF up-regulation initiated 24 h following exposure to RV1b and reached its peak values by 72 h (Figure 1D).

### RV infection upregulates bFGF transcription

We next asked whether the induction of bFGF release reflected a preceding RV-mediated transcriptional activation. To this end, we used semiquantitative PCR, [15] and showed that RV1b infection up-regulated bFGF mRNA at both early (6 h) and late (24 h) time points (Figure 1F) by 2.5- and 3.1-fold, respectively, further suggesting that additional pathways may contribute to the much higher induction observed at the protein level.

### RV-mediated bFGF release positively correlates to RV-induced cytotoxicity

**Figure 2:**
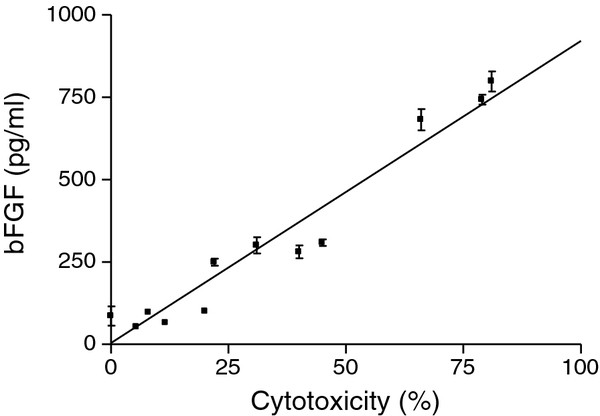
**RV mediated bFGF release is correlated with RV-induced cytotoxicity.** Correlation of released bFGF with RV1b induced cytotoxicity in sparse and dense cultures of BEAS-2B cells (n = 12, p < .0001).

Cell death or injury have been identified as mechanisms of extracellular release of preformed bFGF [25]. On the other hand, we have recently shown that RV-induced epithelial cytotoxicity may become considerable in a compromised, in terms of density, bronchial epithelium, as in the case of asthma [19]. To assess the potential contribution of RV-mediated cytotoxicity on bFGF release by bronchial epithelial cells, we infected the BEAS2B cells with RV1b under conditions of high and low cytotoxicity (sparse and dense cultures, respectively, with sparse cultures roughly corresponding to the partially denuded bronchial airway mucosa in asthma) and estimated the bFGF that was subsequently released. We found that the levels of secreted bFGF significantly correlated with RV-mediated cytotoxicity (Figure 2; R^2^ = 0.91, p < .0001), reaching up to 96-fold induction, implying that cell death-associated bFGF release was an essential component of RV1b-mediated production of this growth factor by bronchial epithelial cells. Similarly, increased cytotoxicity was correlated with increased bFGF secretion by primary bronchial epithelial cells (data not shown).

### VEGF promotes RV-mediated bFGF release

**Figure 3:**
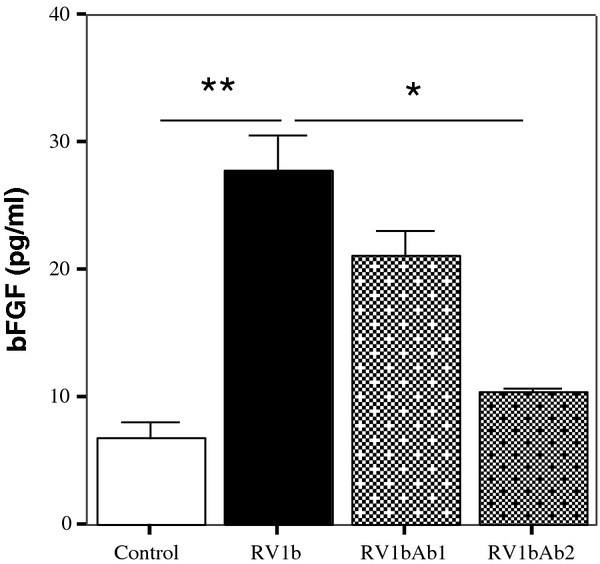
**Anti-VEGF antibody (Ab) suppresses RV mediated bFGF induction.** Partial suppression of RV1b induced bFGF release in the presence of an anti-VEGF Ab in two different concentrations (Ab1 = 0,1 μg/ml and Ab2 = 5 μg/ml). (n = 3) (*p < .01 **p < .001).

Based on the synergistic interaction of VEGF and bFGF in the angiogenesis process and our previous finding that RV infection induces VEGF production by bronchial epithelial cells [15] we sought the possible involvement of VEGF in bFGF release by the bronchial epithelium. A neutralizing anti-human VEGF antibody was added after RV1b infection of epithelial cells resulting in partial inhibition of bFGF release. The inhibition was greater at higher antibody concentrations (Figure 3; p < .01 for 0.1 μg/ml vs p < .001 for 5 μg/ml concentration).

### RV epithelial infection stimulates lung fibroblast proliferation, partly through bFGF

**Figure 4:**
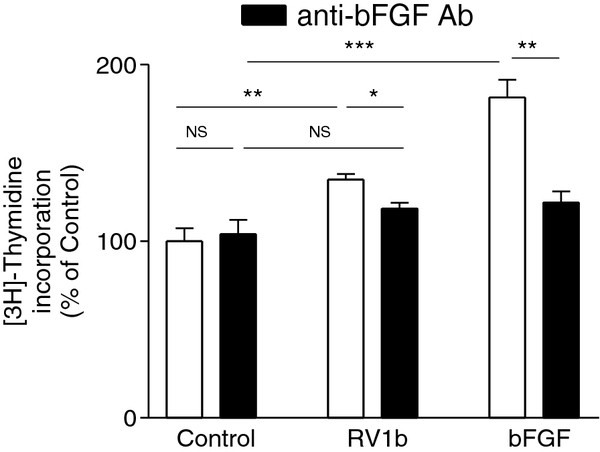
**RV-infected epithelial cells stimulate fibroblast proliferation, partially through bFGF.** Conditioned media (CM) from RV1b infected BEAS-2B cells significantly stimulated the proliferation of quiescent human lung fibroblasts as compared to CM of epithelial cells exposed to HeLa lysate (control). This stimulation was partially suppressed following preincubation of conditioned media with an anti-bFGF neutralizing antibody (Ab). Medium supplemented with 2 ng/ml human recombinant bFGF served as a positive control. (n = 5; NS: non-significant, *p < .01, **p < .001).

To assess the impact of RV-infection of bronchial epithelial cells on stromal cells, UV-irradiated CM from RV1b-infected and control BEAS-2B cultures were applied to serum-starved human CCD19Lu lung fibroblasts. RV1b-infected CM were able to stimulate the proliferation of fibroblasts (34.9 ± 3.1% over control CM; Figure 4; p < .001). These experiments were repeated using primary cultures of normal human fibroblasts with similar results (data not shown). To evaluate the contribution of bFGF in the RV mediated stroma cell proliferation, CM were incubated with neutralizing concentrations of an anti-bFGF antibody. Antibody treatment had no effect on control CM-mediated proliferation, while it significantly suppressed proliferation induced by RV1b-infected CM to 18.6 ± 3.1% over control CM (i.e. 53% of the effect observed in the absence of the antibody; p < .01). Importantly, the residual activity of RV1b-infected CM in the presence of anti-bFGF was not significantly higher than that of the control CM in the presence of the antibody (13.9 ± 3.1%; p = .1295). Similarly, the presence of the antibody significantly inhibited fibroblast proliferation induced by recombinant bFGF (2 ng/ml), serving as a positive control (Figure 4, p < .001).

### RV-infection up-regulates MMP activity released by bronchial epithelial cells

**Figure 5:**
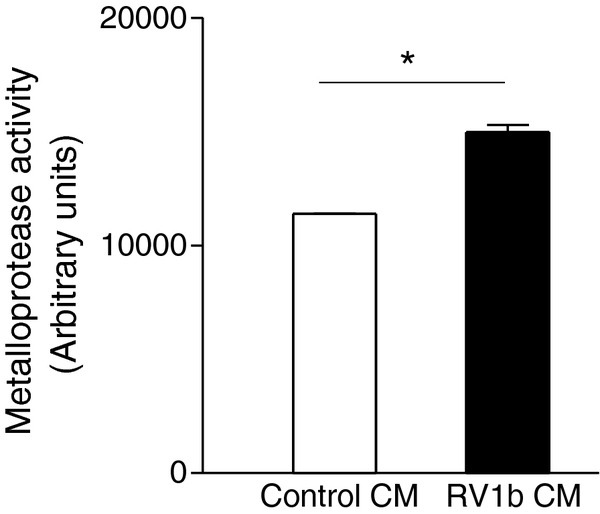
**RV-infected epithelial cells demonstrate an increased matrix metalloproteinase (MMP) activity.** Conditioned media (CM) from RV infected BEAS-2B cells demonstrate an increased MMP activity using an assay detecting MMPs 2, 9, 3 and 1. (n = 3; *p < .05). Arbitrary units correspond to TNO211 fluorescence.

To further examine the potential impact of RV-infection on airway remodeling we measured the total activity of a series of MMPs, in the CM of RV1b-infected epithelial cells. We observed significantly increased MMP activity released in the CM from RV1b-infected BEAS-2B, as compared to control cultures (Figure 5; p < .05).

### RV-induced bronchial epithelial bFGF release is more pronounced in an atopic asthmatic as compared to the non-atopic, non-asthmatic environment

**Figure 6:**
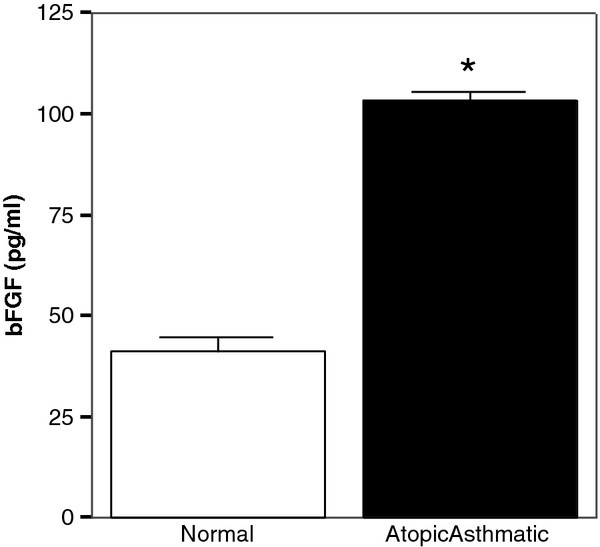
**Exposure to PBMC supernatant affects bFGF release by bronchial epithelial cells.** bFGF release by BEAS-2B cells pre-exposed to supernatants from RV-infected PBMCs isolated from atopic asthmatic (n = 3) or normal (n = 3) subjects and subsequently infected with RV at MOI1 (*P < .001).

We next asked whether the profibrotic response elicited by RV infection is modulated under the influence of an atopic asthmatic environment, as is the case with RV-mediated VEGF induction [15]. To that end, CM of RV1b infected-PBMCs, obtained by control subjects and atopic asthmatic patients, respectively, were added to BEAS-2B and the cells were further stimulated with RV1b, as described [15,18]. CM from RV1b-infected PBMCs rather than purified cytokines were used, to better represent at relevant relative concentrations the complex mixture of mediators resulting from the antiviral immune response [18]. Production of IFN-γ from RV1b-stimulated PBMCs is significantly higher and IL-10 production is lower in normal than in atopic asthmatic subjects [18]. Moreover, RV infection results in a significant upregulation of IL-8 and a statistically insignificant increase of IL-6 production from PBMC, and a parallel statistically insignificant TGF-β1 reduction in both atopic asthmatic and normal subjects [18]. These CM were tested with RT-PCR for the presence of vRNA before being added to BEAS-2B and were found to be negative (data not shown). RV1b infected-PBMCs released low levels of bFGF (median: 5.0 pg/ml). Most importantly, PBMCs from subjects with asthma induced the release of significantly higher amounts (2.5-fold) of bFGF by RV1b-infected BEAS-2B than PBMCs from control subjects (Figure 6; p < .001).

### BFGF levels are up-regulated in the airways during RV-associated asthma exacerbations

**Figure 7:**
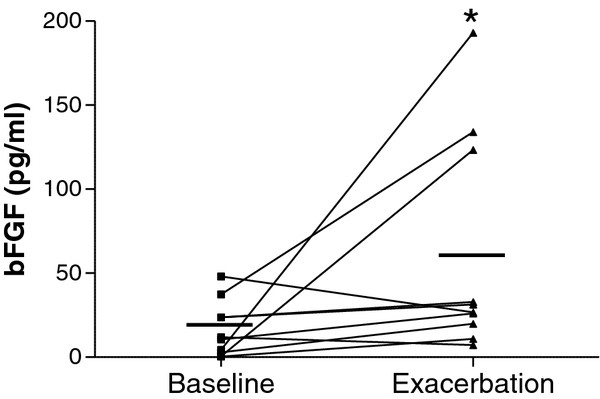
**bFGF levels in human airways during RV-associated asthma exacerbations.** Nasal aspirates were collected from 10 patients with asthma at a stable period (baseline) and subsequently, during a virologically confirmed RV-associated asthma exacerbation. bFGF levels were measured with ELISA. Horizontal bars represent mean values (n=10; *P < .05).

To establish the relevance of the above findings in-vivo, we measured bFGF in nasopharyngeal secretions of children with atopic asthma at baseline and during a subsequent, virologically confirmed RV-associated asthma exacerbation. bFGF levels significantly increased by approximately 5-fold in these patients during exacerbations (Figure 7; 12.9 ± 4.1 pg/mL vs 64.3 ± 21.4 pg/mL; p < .05). Notably, 3 among the 8 children that showed elevated bFGF, demonstrated a dramatic up-regulation of bFGF secretion (32 to 193-fold), whereas the rest 5 children showed a more modest increase (1.3 to 6.6-fold). Yet, no significant differences concerning lower airway symptom scores or changes in lung function were recorded in our study. Overall, RV infection during an asthma exacerbation was indeed associated with increased bFGF secretion in those patients.

## Discussion

This is the first report of RV infection-mediated induction of lung fibroblast proliferation through increased bFGF release by the bronchial epithelium. Moreover, to our knowledge, this is the first time that RV-mediated cytotoxicity is shown to affect the release of a cytokine or growth factor by bronchial epithelial cells. We have previously reported a time-dependent increase in virus titers of RV-infected human bronchial epithelium [12], as well as an RV-mediated bFGF release by such cells, which was suppressed by anti-inflammatory treatment *in vitro*[26]. In this study we extend and link these findings demonstrating that RV-specific bFGF release is time- and dose- dependent and results from both transcriptional up-regulation and release from epithelial cells upon induction of cytotoxicity. Activation of intracellular protein kinases and signaling pathways may be responsible for the transcriptional up-regulation of bFGF, as has been recently demonstrated for RV-mediated VEGF induction [27]; however, such investigations are beyond the scope of this article. On the other hand, it is likely that cell death-mediated pathways prevail over transcriptional regulation, as the 20-fold increase in bFGF levels at 48 h post-infection cannot be fully attributed to the modest 2-fold increase of bFGF mRNA observed at 6 and 24 h post-infection. bFGF is constitutively produced from the epithelium of healthy lungs and its release may be induced by chemical or mechanical insult to these cells [9]. In addition to the release of preformed bFGF from extracellular storage sites, this molecule lacks a conventional secretion signal and is being released by various alternative pathways, including cell death [25]. Epithelial cell desquamation is a central feature of asthmatic airways, while RV-induced cytotoxicity may become considerable in an already compromised epithelium [19], suggesting that such mechanisms may contribute to RV-mediated bFGF release by airway epithelium, being of particular importance in asthma.

Respiratory syncytial virus infection may as well stimulate bFGF release, even in the presence of viral proteins alone [28]. In contrast, RV replication was necessary for enhanced bFGF release as evidenced upon use of heat- or UV-inactivated RV. Despite virus replication, the proportion of RV-infected epithelial cells remains low among exposed cells [29] and differences in the degree of infectivity may account for differences in levels of bFGF release between cell types as reported in the present and our previous study [26].

VEGF seems to play an important role in this process as addition of anti-VEGF antibody resulted in partial suppression of bFGF release. This is in agreement with VEGF-mediated up-regulation of bFGF mRNA reported in other *in vitro* cell injury settings [30]. Also, the use of VEGF receptor inhibitors, both *in vitro* and *in vivo*, blocked bFGF mediated angiogenesis, indicating that the presence of VEGF is crucial in this process [31]. In fact, VEGF and bFGF act synergistically in stimulating angiogenesis and we have demonstrated RV mediated VEGF induction in previous work [15]. In addition, TGF-β1 released by the RV-infected cells [16] may also mediate bFGF release by extracellular deposits, as suggested by studies with human pulmonary fibroblasts [17]. Potential effects on bFGF expression by other cytokines, chemokines and growth factors, which are known (or yet unknown) to show increased production by bronchial epithelial cells following RV infection [13,18,27,32,33] cannot be excluded.

bFGF has been shown to induce cell proliferation in quiescent cultures of human lung fibroblasts [34,35], a finding which we also confirmed (Figure 4). Although bFGF is a milder stimulus for lung fibroblasts than classical mitogens such as EGF and PDGF [35], exposure to conditioned media of RV-infected epithelial cells containing bFGF at much lower levels than our positive control (approx. 10-fold), resulted in a weaker, as expected, but nonetheless significant stimulation of fibroblast proliferation. Notably, we showed that the RV-induced fibroblast proliferation may be exacerbated in an atopic asthmatic environment, further reinforcing its biological significance. Preincubation of RV-infected conditioned medium with an anti-bFGF antibody resulted in a partial, yet considerable suppression of the RV-induced stroma cell proliferation, implying bFGF as a major mediator. Stimulation of proliferation could be also attributed, at least in part, to the presence of other cell-secreted products stabilizing or presenting bFGF to its receptor, or simply acting in synergism [36]. Indeed, several other mediators reported to be produced by bronchial epithelial cells are good candidates as contributors to the remaining fibroproliferative activity of RV infected CM, including TGF-β, IGF-1, PDGF, and endothelin. Accordingly, we have recently observed up-regulation of TGF- β1 activity upon RV-infection [18]. Notably, TGF- β1 presence may act synergistically to further induce bFGF’s proliferative action in the longer term [37], further potentiating the proliferative capacity of bronchial epithelial secretions upon RV infection.

In addition to that, RV infection results in stimulation of MMP activity released by bronchial epithelial cells, in accordance to recent findings [38]. Increased MMP-9 levels have been detected in BAL fluid [39], hypertonic saline-induced sputum [39,40] and serum [41], of asthmatic patients, while sputum [40] and serum [41] levels have been correlated to disease severity. Apart from their well known role in extracellular matrix (ECM) turnover, MMPs of bronchial epithelial origin have been shown to promote fibroblast proliferation [5], suggesting a potent profibrotic effect. Moreover, the MMPs have been shown to activate several secreted cytokines and growth factors [42], and thus their presence in an activated form in the epithelial supernatants could further modulate fibroblast proliferation. Most importantly, pro-MMP9 may potentiate bFGF bioavailability and action [43], thus further contributing to the bFGF-mediated remodeling features of RV infection. Overall, the RV-mediated induction of MMP activity released by the bronchial epithelium can modulate both fibroblast proliferation and collagen turnover, globally affecting airway remodeling.

Upon virus infection both systemic and local immune responses are being elicited, further characterized by a defective Th1 response in atopic asthma [44]. Indeed, Korpi-Steiner et al. recently showed that RV-infected PBMCs produce CXCL10 and CCL2 [32], while we observed higher IL-10 and lower IFN-γ production from RV-stimulated PBMC of atopic asthmatic versus normal subjects [18]. Moreover, RV infection results in a significant increase of IL-8 and a statistically insignificant increase of IL-6 production from PBMC, and a parallel statistically insignificant TGF-β1 reduction in both atopic asthmatic and normal subjects [18]. In addition, macrophages from allergen-sensitized and -challenged mice, but not control mice, express an array of Th2 cytokines following RV infection [45]. Along these lines, RV-induced PBMC responses correlate well with lung function measures in asthma [46]. Accordingly, we investigated the role of atopy and allergy in modulating RV-induced bFGF release by bronchial epithelial cells by pre-exposing the latter to CM, generated by exposure of PBMC to RV. Using this recently described model [18] we have previously demonstrated an enhanced RV-mediated epithelial VEGF induction in the atopic asthmatic context [15]. The here observed enhanced bFGF release under the influence of an “atopic asthmatic” environment may recapitulate the effect of an altered cytokine profile of RV-infected PBMCs from atopic asthmatics [18,44]. For instance, elevated IL-8 release upon RV infection [18] may partly explain the induction of bFGF production by PBMC-exposed bronchial epithelial cells, as this chemokine has been shown to induce both expression and release of bFGF in other settings [47]. Our findings are consistent with the current aspect that altered immune response in asthma may affect airway remodeling [1]. In the same setting, future studies examining the potential effect of mediators released by allergen- or allergen- plus RV-stimulated PBMCs may offer additional insight to the role of virus-allergen interactions on lower airways remodeling.

Our *in vitro* data were confirmed by measuring levels of bFGF released in the airways following RV-induced asthma exacerbations. The elevated bFGF in nasal secretions of asthmatic children may represent a link of viral infections with bFGF and the remodeling process as a whole. Inclusion of asthmatic children with a virus-induced exacerbation attributed to other viruses than RV as well as non-asthmatic children with an upper respiratory tract infection due to RV would offer additional valid comparisons and their absence represents a limitation of our study. The increased concentration of bFGF recorded in the present study could be attributed to increased production by stroma or inflammatory cells, as well as release of preformed protein bound to the ECM. Indeed, both intact and cleaved heparan sulfate proteoglycan (HSPG) have been shown to bind and release bFGF in the asthmatic airways. HSPG distribution differs considerably among asthma patients [9], and HSPG from different patients may respond differentially in releasing bound bFGF. In addition thrombin, whose levels in the airways of asthmatics may differ up to 10-fold, can facilitate bFGF release leading to up to 5-fold differences in bFGF levels [48]. The above could partially attribute the more robust bFGF increase observed in 30% of the asthma patients of this study upon RV infection to differences in ECM composition and response. Nonetheless, we also showed that the bronchial epithelium also takes part in bFGF release in an inducible manner.

An increase of bFGF levels in BAL fluid of asthmatic patients, also relating to increased vascularity in asthmatic airways has been reported [7,8]. Increased bFGF levels in asthmatics as compared to controls even at baseline status may be attributed to delayed viral clearance, which is prominent in atopic patients [49]. Sustained high levels of such growth factors may play a vital role in remodeling and collagen turnover processes.

In summary, we demonstrated that in addition to VEGF production [15,27], RV infection induces bFGF release by the bronchial epithelium. bFGF may thus mediate an additional role of RV infection on asthmatic airway remodeling by stimulating proliferation of stroma cells, accentuated in the context of an atopic environment. These findings further support the notion that in addition to asthma exacerbations, an abnormal response to viral infection may contribute to the persistence of asthma.

## Competing interests

Sebastian L Johnston has received funding from AstraZeneca, Centocor, GlaxoSmithKline (GSK), Pfizer, sanofi pasteur between 2005 & 2012 for studies investigating mechanisms of exacerbations of asthma and/or COPD. Nikolaos G Papadopoulos is the recipient of funding/financial support for speaking at conferences sponsored by Merck and GSK and for research from Astra Zeneca, GSK and Merck. All other authors have no conflict of interest to declare.

## Authors’ contributions

CLS carried out the major part of the experiments and drafted the manuscript. SP participated in the study design, supervised and contributed to the analysis and interpretation of major experiments and helped in finalizing the manuscript. EV participated in a large part of the experiments executed. HP performed and analyzed the experiments on fibroblast proliferation and matrix metalloproteinase activity. ISS participated in the epithelial cell experiments. MG performed the bronchoscopies, during which primary bronchial epithelial cells were sampled. VG participated in the epithelial cell experiments. SV performed bronchoscopies. AGT contributed to *ex vivo* experimentation. PM participated in the peripheral blood mononuclear cells’ experiments. DK contributed to and analyzed the experiments on fibroblast proliferation and matrix metalloproteinase activity. DG contributed to epithelial cell experimentation. SLJ contributed to supervision of *ex vivo* experimentation. NGP conceived the study, participated in its design, data interpretation and coordination. All authors have read and approved the final manuscript.
